# Functional Profiling of Transcription Factor Genes in *Neurospora crassa*

**DOI:** 10.1534/g3.117.043331

**Published:** 2017-07-07

**Authors:** Alexander J. Carrillo, Patrick Schacht, Ilva E. Cabrera, Johnathon Blahut, Loren Prudhomme, Sarah Dietrich, Thomas Bekman, Jennifer Mei, Cristian Carrera, Vivian Chen, Isaiah Clark, Gerardo Fierro, Logan Ganzen, Jose Orellana, Shelby Wise, Kevin Yang, Hui Zhong, Katherine A. Borkovich

**Affiliations:** * Department of Plant Pathology and Microbiology,; † Graduate Program in Microbiology,; ‡ Graduate Program in Genetics, Genomics and Bioinformatics, and; § College of Natural and Agricultural Sciences, University of California, Riverside, California 92521

**Keywords:** filamentous fungi, transcription factors, functional genomics, gene knockouts, transcriptional profiling

## Abstract

Regulation of gene expression by DNA-binding transcription factors is essential for proper control of growth and development in all organisms. In this study, we annotate and characterize growth and developmental phenotypes for transcription factor genes in the model filamentous fungus *Neurospora crassa*. We identified 312 transcription factor genes, corresponding to 3.2% of the protein coding genes in the genome. The largest class was the fungal-specific Zn_2_Cys_6_ (C6) binuclear cluster, with 135 members, followed by the highly conserved C2H2 zinc finger group, with 61 genes. Viable knockout mutants were produced for 273 genes, and complete growth and developmental phenotypic data are available for 242 strains, with 64% possessing at least one defect. The most prominent defect observed was in growth of basal hyphae (43% of mutants analyzed), followed by asexual sporulation (38%), and the various stages of sexual development (19%). Two growth or developmental defects were observed for 21% of the mutants, while 8% were defective in all three major phenotypes tested. Analysis of available mRNA expression data for a time course of sexual development revealed mutants with sexual phenotypes that correlate with transcription factor transcript abundance in wild type. Inspection of this data also implicated cryptic roles in sexual development for several cotranscribed transcription factor genes that do not produce a phenotype when mutated.

Transcriptional control by sequence-specific DNA-binding proteins is a major regulatory mechanism in all organisms (Weirauch and Hughes 2011). It has been estimated that there are >90 types of transcription factors in eukaryotes (Weirauch and Hughes 2011). Major structural classes of eukaryotic transcription factors include zinc coordinating (C2H2 and C4 zinc fingers and Zn_2_Cys_6_/C6 zinc clusters), helix-turn-helix (HTH), β-scaffold, and proteins with basic domains (basic leucine zipper/bZIP and basic helix-loop-helix/bHLH) (Weirauch and Hughes 2011). Although zinc finger proteins predominate in eukaryotic genomes, the HTH group is the most widely conserved transcription factor group across evolution, as it comprises the majority of transcription factors in bacterial and archaeal genomes (Aravind *et al.* 2005; Weirauch and Hughes 2011).

Early studies proposed that transcription factors were 0.5–8% of the gene content in the genome, and that the number of genes was roughly proportional to the size of the genome (Levine and Tjian 2003). In recent years, the widespread availability of genome sequences has made it possible to annotate the actual number of transcription factor genes from numerous eukaryotic species. There are estimated to be ∼19,000 protein-coding genes in humans (Ezkurdia *et al.* 2014). Various groups have annotated DNA-binding transcription factor genes over the years and identified >1300, with most discrepancies resulting from transcript variants for some genes (Vaquerizas *et al.* 2009; Wingender *et al.* 2013). For example, Vaquerizas *et al.* (2009) annotated 1391 transcription factors, comprising 7.3% of total protein coding genes in the human genome. They found that three classes of transcription factor genes predominate in humans: C2H2 zinc finger (675 genes), homeodomain (257 genes), and bHLH (87 genes). A later study identified 1558 transcription factor genes in the human genome (8.2% of protein-coding genes), comprising 111 families in 40 classes and 10 superclasses (Wingender *et al.* 2013). C2H2 transcription factors were reported as the largest structural class at 53%, followed by HTH at 26%, and basic domain transcription factors at 11%. In the fruit fly *Drosophila melanogaster*, there are 708 genes out of 13,929 protein-coding genes (5%) that encode predicted transcription factors (Gramates *et al.* 2017; Hammonds *et al.* 2013). C2H2 zinc fingers comprise the largest group with 255 members, followed by homeobox (101 genes), and HLH (58 genes) proteins. The model plant *Arabidopsis thaliana* has 1717 predicted transcription factors out of the 27,029 protein-coding genes in the genome (6.4% of genes) (Jin *et al.* 2015; Swarbreck *et al.* 2008). These genes are organized in 58 families, with the largest classes being HLH (225 genes), MYB (168 genes), AP2/ERF (139 genes), the plant-specific NAC family (138 genes), bZIP (127 genes), and C2H2 zinc fingers (116 genes).

Fungi contain several types of transcription factors not found in animals or plants, including the Zn_2_Cys_6_ (C6) zinc cluster, APSES (*Neurospora crassa* ASM1, *Saccharomyces cerevisiae* Phd1p, *Aspergillus nidulans* StuA, *Candida albicans* Efg1, and *S. cerevisiae* Sok2p), copper fist, STE (sterile) and Velvet classes (Iyer *et al.* 2002; Weirauch and Hughes 2011; Ahmed *et al.* 2013; Hughes and de Boer 2013). In the model yeast *S. cerevisiae*, there are currently 301 predicted transcription factors out of the 6604 protein-coding genes in the genome [4.6% of genes; (de Boer and Hughes 2012; Hughes and de Boer 2013; Engel *et al.* 2014)]. The largest class of transcription factors in the yeast genome is the C6 zinc cluster (57 genes), followed by C2H2 zinc fingers (41 proteins), bZIP (15 proteins), homeodomain (12 proteins), GATA factors (10 proteins), and bHLH (eight proteins) (Hughes and de Boer 2013).

*N. crassa* is multicellular fungus and eukaryotic model system that has been studied for >75 yr (Borkovich *et al.* 2004; Selker 2011). A genome-wide project has resulted in a nearly complete gene knockout mutant collection for the almost 10,000 genes in the genome (Colot *et al.* 2006; Dunlap *et al.* 2007; Park *et al.* 2011a). We had previously annotated 182 transcription factor genes in the *N. crassa* genome (Borkovich *et al.* 2004), and attempted mutation of 103 of these genes (Colot *et al.* 2006). We were able to isolate viable knockout mutants for 99 genes, and these were analyzed for growth and developmental phenotypes (Colot *et al.* 2006). The results demonstrated that 43% of the transcription factor mutants had at least one phenotype, with greater than half of these possessing multiple defects (Colot *et al.* 2006).

In this study, we have cataloged functions for the majority of transcription factors in a filamentous fungus. We annotate 130 more transcription factor genes in the genome, bringing the total number in *N. crassa* to 312. We combine the data from our earlier study (Colot *et al.* 2006) with that for the additional genes, presenting phenotypes for a total of 242 available viable knockout mutants. We mine an RNAseq dataset for expression of the 312 genes during different phases of sexual development. Our results demonstrate that the majority of genes yield at least one phenotype, and that multiple transcription factors are required to control different aspects of growth and development in *N. crassa*.

## Materials and Methods

### Transcription factor gene annotation

Data from the 182 predicted transcription factor genes identified previously (Borkovich *et al.* 2004; Colot *et al.* 2006) were included in this study. This list was augmented with additional genes obtained through searches at the FungiDB or Broad Institute Neurospora Genome databases (*Neurospora crassa* Genome Project 2015; Stajich *et al.* 2012), the CIS-BP database at http://cisbp.ccbr.utoronto.ca (Weirauch *et al.* 2014), and from a list that was generously shared with us by Luis Larrondo (Pontificia Catholic University of Chile, Santiago, Chile). All entries were evaluated in our laboratory using BLAST homology searches (Altschul *et al.* 1990), followed by analysis of PFAM domains using Interpro (Hunter *et al.* 2012). Genes encoding domain(s) established to be DNA binding transcription factors, and with *E*-values <10^−5^, were included in our final list. An exception to the DNA binding requirement was made for WD40 domain-containing transcriptional adapters, in light of their importance to growth, development, and environmental sensing in fungi.

### Media and mutant construction

Vogel’s minimal medium (VM) (Vogel 1964) was used to support asexual growth and development, while synthetic crossing medium (SCM) (Westergaard and Mitchell 1947) was utilized to assess sexual development. Formation of tight colonies on plates was facilitated by growth on sorbose-containing medium plates (Davis and deSerres 1970). Hygromycin B (Calbiochem, San Diego, CA) was used at a concentration of 200 μg/ml in media where indicated. Inoculations were performed using macroconidia harvested from VM agar slants (Davis and deSerres 1970).

Wild-type strains ORS-SL6a (FGSC 4200; *mat a*) and 74-OR23-IVA (FGSC 2489; *mat A*) were obtained from the Fungal Genetics Stock Center (FGSC; Kansas State University, Manhattan, KS). Available transcription factor mutants (homokaryons or heterokaryons) were obtained from the FGSC, or produced in our laboratory (see Supplemental Material, Table S1 for strain and phenotype information). All *N. crassa* gene numbers are preceded by the prefix “NCU.” We attempted purification of homokaryons from 28 heterokaryotic transformants that were available from the FGSC or our laboratory stocks (Table S1) using sexual crosses (as described in Colot *et al.* 2006) or streaking of macroconidia in the vegetative phase (Davis and deSerres 1970). All putative homokaryons were checked for the presence of the knockout cassette using *hph* and gene flank-specific primers by polymerase chain reaction as described (Ghosh *et al.* 2014). Those strains purified through streaking of macroconidia were also screened for the absence of the wild-type open reading frame using gene-specific primers. This step was not needed for the cross progeny, since the ascospore meiotic products are homokaryons. We were able to successfully purify 15 mutants using this approach (Table S1). Two additional mutants (NCU07430 /*mad-1* and NCU09496 ) were purified using this method, but were inviable after storage (Table S1). Another seven mutants were removed due to the observation in another study that the strains carry a secondary mutation that results in female sterility (Fu *et al.* 2011). In summary, a total of 70 mutants were not available for analysis at the time of this study, either due to failures in construction of knockout cassettes; changes in gene annotation that made the knockout mutant unusable, or resulted in the knockout mutant not being made; incorrect insertion of knockout cassettes in the genome; the inability to purify homokaryotic mutants from transformants, secondary mutations; or because the original purified knockout mutant was inviable after storage (Table S1). This left us with 242 viable homokaryotic mutants for study.

### Phenotypic analysis

The 242 viable mutants were analyzed for phenotypes using methods described in Cabrera *et al.* (2015), Ghosh *et al.* (2014), Park *et al.* (2011b), and Turner (2011). Data were obtained from the Broad Institute *Neurospora* website (*Neurospora crassa* Genome Project 2015) or from experiments performed for this study. Some of the data at the Broad Institute website was previously published (Colot *et al.* 2006). The combined data are presented in Table S1. We have omitted measurements of pigmentation and aerial hyphae height on yeast extract-containing medium from our analysis (Cabrera *et al.* 2015). Near-isogenic wild type strains FGSC4200 and/or FGSC2489 were used as a control for all determinations. Race tubes made of glass or prepared from disposable plastic pipets were used for quantitative analysis of hyphal growth rates (Dharmananda and Feldman 1979; White and Woodward 1995). Multiple race tubes were analyzed for each mutant, with a minimum of four independent replicates with *R*^2^ > 0.95 used to obtain the average growth rate. The wild-type growth rate range for data obtained from Colot *et al.* (2006) and the Broad Institute *Neurospora* Database was 70–85 mm/d, while that from this study was 75–85 mm/d (see also legend in Table S1). Binned data from Colot *et al.* (2006) and/or the Broad Database were averaged to allow comparison to actual growth rate measurements.

Aerial hyphae height was assessed after incubation of 2-ml VM standing liquid cultures for 3 d in the dark at room temperature, with at least six replicates/strain. Mutants that displayed a growth rate <70 or >85 mm/d, or an aerial hyphae height measurement <30 or >45 mm (over 3 d), were considered significantly different than wild type (Table S1). Macroconidia production was qualitatively assessed by visual inspection of VM agar slants incubated for 3 d in the dark at 25°, followed by 4 d in light at room temperature, with at least four replicates/strain. Three stages of sexual development were qualitatively analyzed using SCM agar slants cultured in constant light at room temperature: formation of protoperithecia after 7 d; development of perithecia from protoperithecia 7 d after fertilization with opposite mating type wild-type conidia; and ascospore shooting from mature perithecia 14 d after fertilization. The quantity and size of protoperithecia, perithecia, and ascospores were observed using a S8APO stereomicroscope with a DFC280 digital camera (Leica Microsystems, Buffalo Grove, IL), or an Olympus SZX9 stereomicroscope with a C-4040 digital camera (Olympus, Lake Success, NY). At least four replicates were analyzed for each strain.

### Clustering of transcription factor gene expression data and heatmap generation

A sexual development time course RNAseq dataset (Wang *et al.* 2014) was mined for expression of the 284 transcription factor genes essentially as described (Cabrera *et al.* 2015). To allow visualization of the expression data, heat maps were produced using pheatmap (V1.0.2) (Kolde 2015) in R V3.1.1; (R Development Core Team 2014). The scaling function in pheatmap was used to normalize expression data, with the reads per kilobase of transcript per million mapped reads (RPKM) values at each time point for a given gene normalized to the RPKM at the time point with lowest expression for that gene.

### Naming of transcription factor genes

In keeping with the *N. crassa* convention, and the naming used in our previous study (Colot *et al.* 2006), unnamed transcription factor genes whose mutations revealed phenotypes received names that reflected the defects. Strains showing abnormalities in all three phenotypes, basal hyphal extension during vegetative growth, asexual development, and sexual development are known as *all development altered* (*ada*). Strains showing altered hyphal growth and asexual development are named as *vegetative asexual development* (*vad*). Mutants with normal hyphal growth but altered sexual and vegetative development are referred to as *sexual and vegetative development* (*svd*), while those with defects in vegetative hyphal growth and sexual development are called *vegetative and sexual development* (*vsd*). Strains showing slower basal hyphal extension, but normal asexual and sexual development are known as *colonial* (*col*) or *slower growth rate* (*sgr*), while mutants with hyphal growth rates greater than wild type are named *faster growth rate* (*fgr*). Mutants with longer aerial hyphae than wild type are named *long aerial hyphae* (*lah*), while those with shorter aerial hyphae are called *short aerial hyphae* (*sah*). Genes whose loss results in a block in female fertility are designated female fertility (*ff*), while those with more subtle sexual cycle defects are known as *defective sexual development* (*dsd*). Mutants that produce submerged perithecia are named as *sub*, while those with defective beaks are referred to as *bek*.

### Data availability

*N. crassa* knockout mutants and wild-type strains are available upon request. File S1 contains detailed descriptions of Supplemental Figures. Table S1 contains genotypes and phenotypes for each mutant. Sequence data are available at the GenBank and FungiDB databases, and the NCU accession numbers for each transcription factor gene are listed in Table S1.

## Results

### Annotation of additional transcription factor genes in the *N. crassa* genome

**Figure 1:**
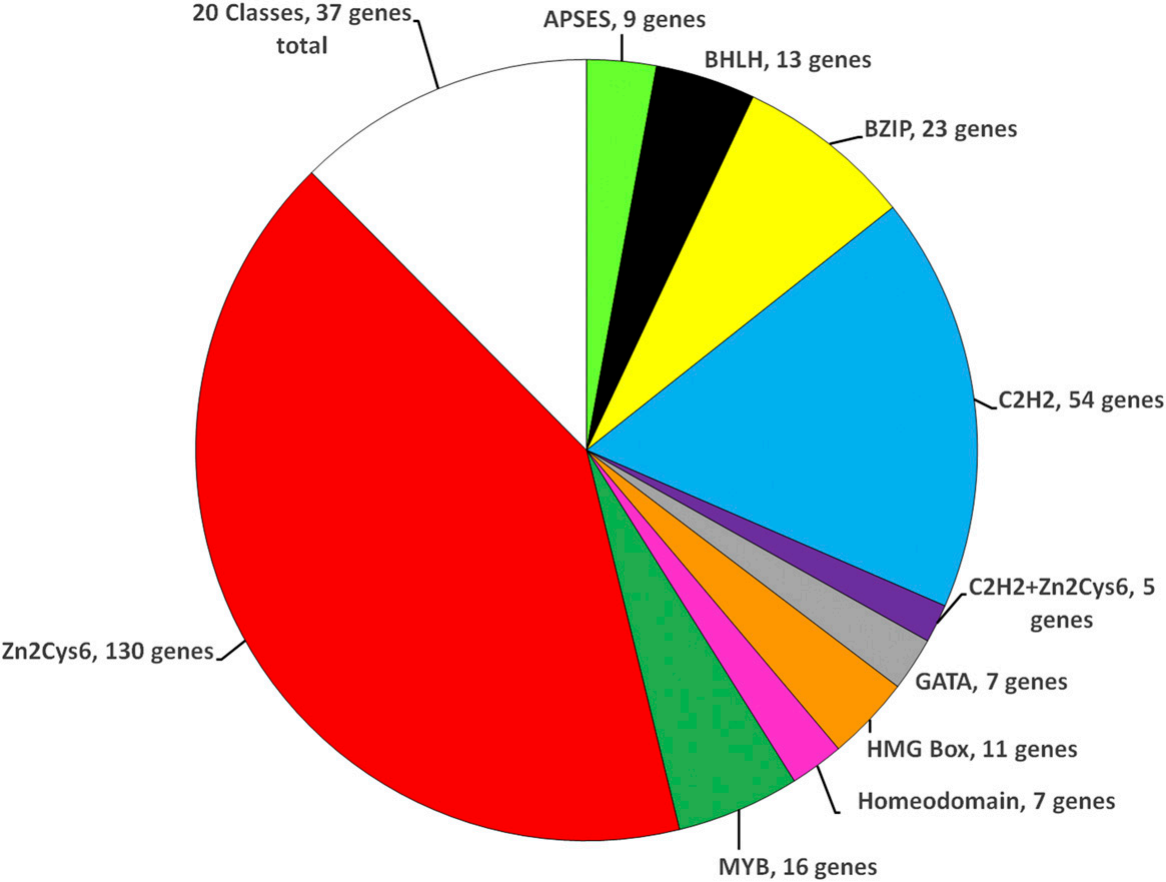
Relative distribution of *N. crassa* transcription factor genes into major classes. Each “slice” of the pie represents the fraction of mutants with the indicated domain. The number of mutants with each domain is indicated. The MISC (Miscellaneous) group includes the 20 domain classes with four or fewer members (see Table 1).

In this study, we annotated an additional 130 transcription factor genes in *N. crassa*, bringing the total number to 312 (Table 1 and Table S1). This corresponds to 3.2% of the 9760 protein-coding genes in the genome (Stajich *et al.* 2012), a proportion that is comparable to *S. cerevisiae*. We identified 25 classes of transcription factors with a single domain, and seven groupings with two distinct domains (Figure 1 and Table 1). The largest group of transcription factors is the C6 binuclear cluster, with 130 genes having this as the only domain (42% of the transcription factors), and another five genes possessing this domain in combination with a second motif (135 genes total; 43% of the transcription factors) (Table 1). This is more than twice the number identified in *S. cerevisiae* (57), despite both organisms having a similar total number of transcription factors (Hughes and de Boer 2013).

**Table 1:** Phenotype summary

TF Class	Total	Genes with Complete Data	Genes with Complete Data (%)	Genes with Data with Phenotype	Genes with Data with Phenotype (%)	Growth	Asexual	Sexual	Growth + Asexual	Growth + Sexual	Asexual + Sexual	All
Single domain TFs												
APSES	9	7	78	3	43	1			1	1		
ARID/BRIGHT	2	2	100	2	100	1						1
AT hook	2	0	0	0								
BHLH	13	12	92	10	83	5		1	2	1		1
BZIP	23	20	87	13	65	3	1		5	3		1
C2H2	54	48	89	30	63	9	7	2	4	2	2	4
CAAT box/CBF	4	3	75	2	67	1						1
Copper fist	2	0	0	0								
CP2	1	1	100	1	100				1			
Far1	3	1	33	1	100		1					
Forkhead	3	3	100	2	67	1				1		
GATA	7	6	86	6	100		2		3	1		
HMG box	11	3	27	2	67						1	1
Homeodomain	7	7	100	5	71	1	1	2	1			
HSF	3	2	67	2	100				2			
MADS-box	2	0	0									
MATalpha1	1	0	0									
MBF	1	0	0									
MYB	16	12	75	11	92	2	1	1	1	2	1	3
NDT80	3	3	100	3	100	1		1	1			
NFX	1	0	0									
RFX	1	1	100	0	0							
Velvet	1	0	0									
WD40	3	3	100	3	100	1						2
Zn2Cys6	130	101	78	53	52	14	19	2	13	1	1	3
Multiple domain TFs												
ARID + APSES	1	1	100	1	100							1
ARID + MYB	1	1	100	1	100						1	
C2H2 + APSES	1	1	100	0								
C2H2+ STE-like	1	1	100	1	100							1
C2H2 + Zn2Cys6	5	3	60	2	67		1	1				
Total	312	242	77.56	154	63.64	40	33	10	34	12	6	19

The second largest group in *N. crassa* is the C2H2 zinc finger, with 54 genes possessing only C2H2, and seven also containing a second domain, for a total of 61 genes (20% of transcription factors)—a number greater than observed for *S. cerevisiae* [41 genes; (Hughes and de Boer 2013)]. The third, fourth, and fifth largest groups of transcription factors in *N. crassa* are the bZIP (23 genes; 7.4% of total), MYB (16 genes with single domain; one gene with two domains; 5.4% of total), and bHLH (13 genes; 4.2% of total) (Figure 1 and Table 1). *N. crassa* has significantly more single domain bZIP (23 *vs.* 15), bHLH (13 *vs.* 8), and MYB factors (16 *vs.* 6) (Hughes and de Boer 2013). Finally, we identified a total of nine genes with two distinct domains, corresponding to 2.9% of the annotated transcription factors (Table 1). As noted above, the most common domain in the genes with two domains was the C2H2 zinc finger, with seven genes (Table 1).

### Mutant production and analysis of hyphal growth phenotypes

All mutants used in this study were produced during the *Neurospora Genome Project* (Dunlap *et al.* 2007), which included a high throughput gene knockout project (Colot *et al.* 2006; Dunlap *et al.* 2007; Collopy *et al.* 2010; Park *et al.* 2011a). Each mutant carries a gene replacement mutation, with insertion of a hygromycin resistance gene cassette in place of the open reading frame (Colot *et al.* 2006). There were instances where gene annotation changes or issues with primer, knockout cassette or strain construction rendered some mutants unusable, or unable to be produced (see *Materials and Methods* and Table S1 for details). In the end, we were able to assemble a group of 242 viable mutants for phenotypic analysis (78% of the total genes; Table 1). The mutants included homokaryons for the four putative essential genes described in our earlier study (Colot *et al.* 2006), which were isolated by streak-purifying in vegetative phase or by screening many ascospores: NCU00340/*pp-1*, *asl-1*/NCU01345, *ts* (formerly *asl-2*; NCU01459), and *cpc-1*/NCU04050.

We analyzed the 242 viable mutants for an array of growth and developmental phenotypes, beginning with the linear growth rate on minimal medium (Colot *et al.* 2006; Turner 2011). *N. crassa* grows by polar extension, branching, and anastomosis (fusion) of tube-like structures called hyphae to form the web-like multicellular structure termed the mycelium (reviewed in Springer 1993; Borkovich *et al.* 2004; Glass *et al.* 2004; Selker 2011). Hyphae contain incomplete crosswalls (septa) that separate cellular compartments, but allow distribution of metabolites and organelles throughout the mycelium (Glass *et al.* 2004).

**Figure 2:**
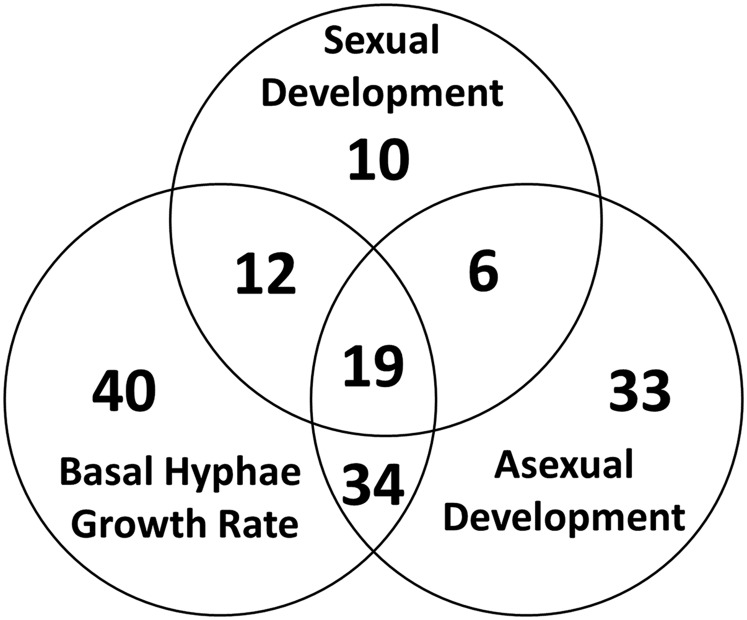
Venn diagram summary of mutants with growth and developmental phenotypes. The total number of mutants with the indicated phenotype or combination of phenotypes is shown in each lobe of the Venn diagram.

We assessed the hyphal growth rate of the mutants on minimal medium using race tubes (Dharmananda and Feldman 1979; White and Woodward 1995). The results demonstrated that 105 mutants had growth rates significantly different from wild type (43% of viable mutants), and altered growth rate was the predominant phenotype in the transcription factor mutants (Figure 2 and Table 1). Consistent with its size, the transcription factor class (present alone or with a second domain) with the largest absolute number of mutants with hyphal growth defects was the C6 family (31/103 viable mutants; 30%; Figure S1 in File S1 and Table 1). The C2H2 group had a slightly higher proportion of mutants with hyphal growth phenotypes (19/51 mutants; 37%; Figure S1 in File S1 and Table 1). However, there were several transcription factor classes with 65–100% of the mutants exhibiting a hyphal growth phenotype. For example, 100% of the mutants with CP2, HSF, and WD40 domains had a hyphal growth defect. Similarly, 10/12 BHLH genes yielded phenotypic data, and nine of these had a growth rate phenotype (75% of viable mutants; Figure S1 in File S1). For genes with the MYB domain, 13/17 were represented as viable mutants, and 8/13 (62%) had a hyphal growth defect (Figure S1 in File S1 and Table 1).

**Figure 3:**
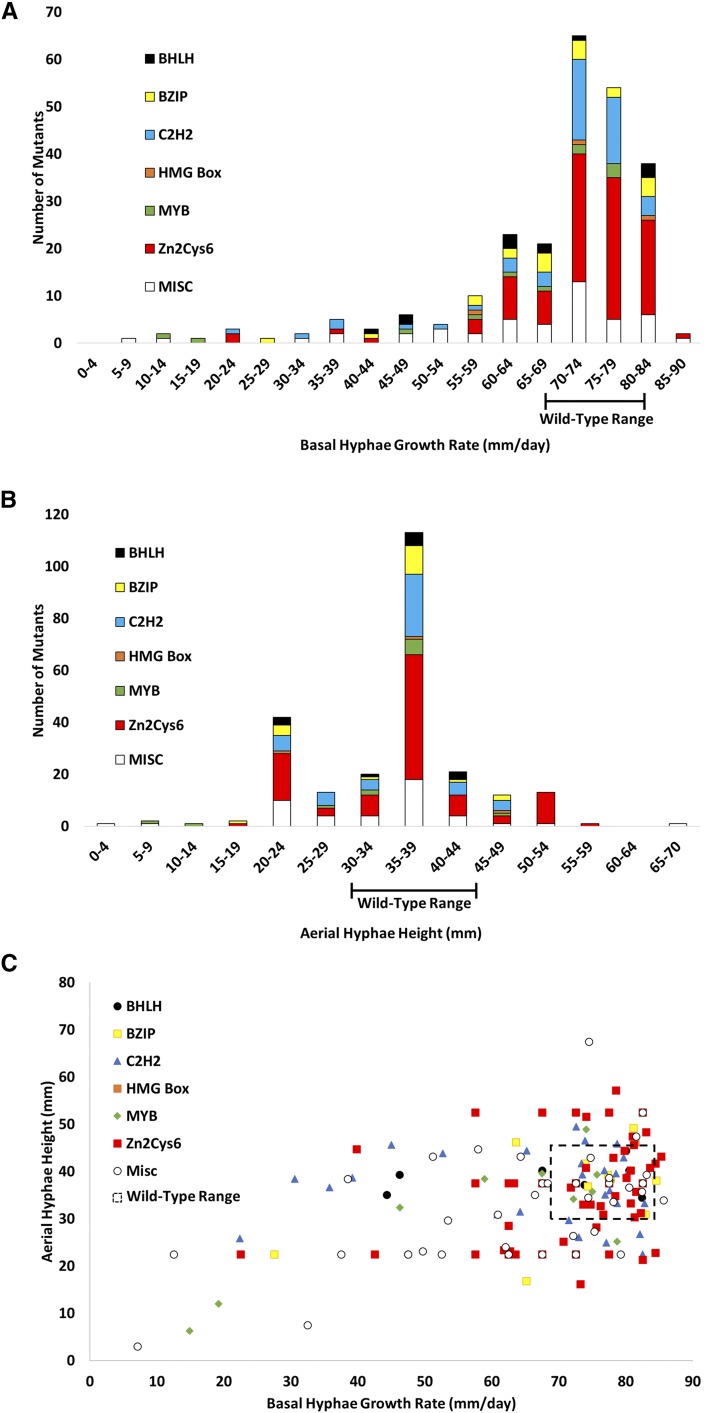
Basal hyphae growth rate and aerial hyphae height phenotypes for mutants in different transcription factor classes. (A) Basal hyphae growth rate. Race tubes containing VM agar medium were inoculated with transcription factor mutants and incubated in the dark at 25°. The growth front was marked after overnight growth (*t* = 0), and then marked twice/day over the course of 2–3 d. Growth rate was determined using linear regression analysis (see *Materials and Methods* for details). Mutants were grouped in bins, as shown. The range of measurements for wild type is indicated on the *x*-axis. (B) Aerial hyphae height. Standing liquid VM tube cultures were inoculated with mutants and incubated statically for 3 d in the dark at 25°, after which the height of aerial hyphae was measured. Values were obtained using at least six replicates and are presented as described in (A). (C) Comparison between aerial hyphae height and basal hyphae growth rate for all mutants. The data from (A) and (B) were plotted. Mutants that fall within the range of wild type values are enclosed by the dashed-line rectangle.

Less than half of the mutants possessed a growth rate significantly slower than wild type (range of 70–84 mm/d; Figure 3A and Table S1). Several mutants displayed a growth rate within the 60–64 and 65–69 mm/d increments, while far fewer were found in the groups with the slowest growth rates (Figure 3A). The <59 mm/d groups contained representation from several transcription factor classes, with loss of the WD40 gene, *rco*-1/NCU06205 (Yamashiro *et al.* 1996), resulting in the slowest growth rate observed in our study (Figure 3A and Table S1). Only two mutants grew faster than wild type; one lacking the C6 zinc finger gene *fgr-1*/NCU10597, and one deleted for the APSES gene *vsd-5*/NCU07587 (Figure 3A and Table S1).

### Asexual development defects

*N. crassa* produces two types of asexual spores, microconidia, and macroconidia (Springer and Yanofsky 1989; Springer 1993; Bistis *et al.* 2003). Microconidia are small, uninucleate, and relatively nonabundant, and are difficult to observe under laboratory conditions (Springer 1993; Bistis *et al.* 2003). In contrast, the multinucleated macroconidia are produced profusely in wild-type cultures (Springer and Yanofsky 1989). The macroconidiation (hereafter referred to as conidiation) pathway is regulated by oxygen/reactive oxygen species, carbon and nitrogen availability, high temperatures, blue light, and the circadian rhythm (Springer and Yanofsky 1989; Hansberg *et al.* 1993; Springer 1993; Greenwald *et al.* 2010; Baker *et al.* 2012). Due to these multiple layers of regulation, wild type does not form conidia in submerged liquid cultures unless subjected to heat shock, carbon stress, or nitrogen stress (Cortat and Turian 1974; That and Turian 1978; Plesofsky-Vig *et al.* 1983; Guignard *et al.* 1984; Madi *et al.* 1997). The conidiation pathway begins with adhesion of basal hyphae, followed by growth of aerial hyphae that rise perpendicular to the growth surface (Springer and Yanofsky 1989; Hansberg *et al.* 1993; Springer 1993). Aerial hyphae form branches, with some transitioning from hyphal growth to apical budding as constrictions form between cellular compartments at the hyphal tip (Springer and Yanofsky 1989). With time, the constrictions tighten to separate the mature multinucleated conidia.

Several transcriptional regulatory proteins have been identified that regulate aspects of conidiation in *N. crassa*. For example, mutants lacking the transcription factor *fluffy* [*fl*/NCU08726; (Bailey and Ebbole 1998)] and the transcriptional adapter *rco-1*/NCU06205 (Yamashiro *et al.* 1996) are blocked at distinct points in the process. Other work has demonstrated transcription factors that influence conidiation under different environmental conditions or the extent of conidiation, including *vad-5*/NCU06799 (Sun *et al.* 2012), *chc-1*/NCU00749 (Sun *et al.* 2011) and *hsf-2*/NCU08480 (Thompson *et al.* 2008). The *white collar* complex (WCC) containing the transcription factors *wc-1*/NCU02356 and *wc-2/*NCU00902 has been demonstrated to directly control expression of 24 transcription factor genes under blue light regulation (Smith *et al.* 2010; Chen *et al.* 2009).

In this study, we identified 92 transcription factor mutants (38% of total mutants) with a defect in aerial hyphae height and/or conidia production (Figure 2 and Table 1). Of these 92 strains, the majority (87; 95%) displayed an aerial hyphae phenotype, either singly (69 mutants; 75%) or in combination with a conidiation defect (18 mutants; 20%) (Table S1). A total of five mutants (5%) had a conidiation defect with normal aerial hyphae height (Table S1). Similar to what was observed for hyphal growth rate, and in keeping with the large number of genes in this family, the C6 transcription factor class had the largest number of mutants with a phenotype (37; 36% of all mutants with C6 domain; Figure S1 in File S1 and Table 1). However, other domain classes had a larger proportion of viable mutants with an asexual development defect, including HSF (2/2; 100%), GATA (5/7; 71%), WD40 (2/3; 67%), HMG-box (2/3; 67%), and MYB (7/13; 54%) (Table 1).

Of the mutants with an aerial hyphae height defect, the majority had shorter aerial hyphae (60 mutants; 65%; Figure 3B and Table S1). However, in comparison to the hyphal growth rate analysis described above, there were more mutants with aerial hyphae height greater than wild type (27 mutants; 29%; Figure 3, A and B and Table S1). The large C6 family dominated the mutants at both ends of the aerial hyphae height spectrum, while other groups appeared in only one section. For example, the Far1 (*tah-9*/NCU06551 ) and CP2 (*csp-2*/NCU06095 ) class transcription factor mutants were all taller than wild type, with the *csp-2* mutant the tallest in our study (Table S1). The other end of the spectrum includes mutants lacking genes with two transcription factor domains: the C2H2 + STE mutant *pp-1*/NCU00340 , the ARID + MYB mutant *svd-1*/NCU04079 and the ARID + APSES mutant *ada-9*/NCU01238, which were all shorter than wild type (Table S1). Similar to observations for hyphal growth rate, the WD40 mutant *rco-1*/NCU06205 had the shortest aerial hyphae overall (Table S1).

Inspection of the qualitative data for conidia production in agar slants showed that a total of 23 mutants was affected (Table S1). Three mutants were increased (lacking the C2H2 *cre-1*/NCU08807, C2H2+STE *pp-1*/NCU00340, and MYB *ada-22*/NCU08003), and 16 reduced, relative to wild type (Table S1). Three mutants (lacking the C6 gene *fl*/NCU08726, the BZIP *ada-1*/NCU00499, and the ARID/BRIGHT *ada-20*/NCU05891) did not form any conidia (Table S1), and the *kal-1*/NCU03593 homeodomain mutant had an abnormal conidiation pattern (Colot *et al.* 2006). The mutant lacking the MYB gene *rcn-1*/NCU07834 had a conidiation defect as its only phenotype (Table S1).

### Sexual development phenotypes

**Figure 4:**
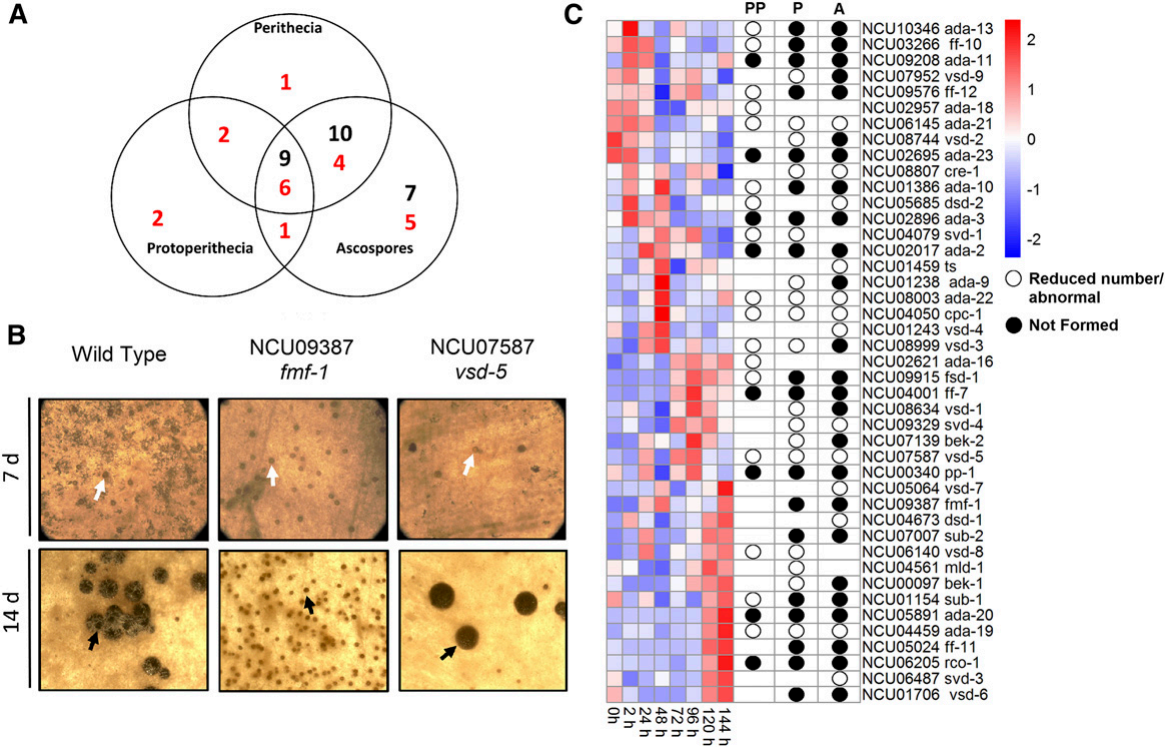
Transcription factor mutants with defects in sexual development. (A) Summary of sexual cycle phenotypes. The total number of mutants with the indicated sexual cycle phenotype is shown in each lobe of the Venn diagram. Mutants with reduced number or abnormal phenotypes (red font) or complete block (black font) are scored according to the stage of their earliest defect. (B) Examples of mutants with different sexual cycle phenotypes. All strains were cultured on synthetic crossing medium plates in constant light at room temperature for 7 d to facilitate development of protoperithecia (top panels). Cultures were then fertilized using macroconidia from a wild-type strain of opposite mating type. Plates were then incubated under the same conditions for a further 7 d to allow production of fertilized perithecia and beak development (bottom panels). Images of protoperithecia were captured using an Olympus SZX9 stereomicroscope with a C-4040 digital camera, while perithecia were photographed using a S8APO stereomicroscope with a DFC280 digital camera. White arrows indicate unfertilized protoperithecia (top panels), while black arrows show protoperithecia (*fmf-1*) or perithecia (wild type and *vsd-5* mutant) 7 d after fertilization (bottom panels). The beak at the tip of a perithecium can be seen as the darkened circular area above the black arrow in wild type (bottom left panel), while *vsd-5* mutant perithecia lack this structure (bottom right panel). (C) Clustering of mRNA expression data for *N. crassa* transcription factors during a time course of sexual development. Left side of figure: RNAseq data were obtained from Wang *et al.* (2014). Expression data for 43 of the 47 transcription factor genes with a sexual cycle phenotype were contained in the data set. Clustering analysis and heatmap generation were performed as described in the *Materials and Methods*. Red shading denotes greater levels of expression, while blue indicates lower expression. The numbers along the left side of the figure indicate groupings (1–6) based on similar patterns of expression during sexual development. The table on the right side of the figure is a phenotype summary for the mutants lacking each transcription factor. The open circles denote that the indicated structure is abnormal or that a reduced number is formed, while closed circles show that the indicated structure is not formed. The absence of a circle indicates there was no defect observed. PP, Protoperithecia; *P*, Perithecia; A, Ascospores.

*N. crassa* is a hermaphrodite, in that a single colony produces female and male gametes. However, *N. crassa* is not self-fertile, requiring that the female and male cells be of opposite mating type [*mat A* and *mat a*; (Raju 1992)]. Nitrogen starvation induces differentiation of female reproductive structures (protoperithecia) (Raju 1992) from the basal hyphae of the mycelium (see wild-type image in Figure 4B). Protoperithecial development begins with coiling, extension, adhesion, and then septation and branching of hyphae to form a coil (Lord and Read 2011). Enveloping hyphae encircle the coil, and then grow and branch to form the protoperithecium. Mating occurs when male-receptive hyphae (trichogynes) from the mature female structure grows toward a male (typically an asexual spore; macro- or microconidium) of opposite mating type in a process involving a pheromone response (Bistis 1983). After fusion of the trichogyne and conidium, the nuclei from the male and female divide synchronously to form the ascogenous hyphae within the developing perithecium (Raju 1992) (see wild-type image in Figure 4B). Nuclear fusion and meiosis takes place in a specialized cell type (crozier). The perithecium enlarges, melanizes, and forms a beak at the tip as the meiotic progeny (ascospores) mature within. Ascospores are then forcibly ejected from a hole (ostiole) in the tip of the beak (Raju 1992). The entire sexual cycle can be completed within 2.5 wk (Davis and deSerres 1970). Blue light is an important environmental cue for sexual development, regulating the abundance of protoperithecia.

In the budding yeast *S. cerevisiae*, only a few genes necessary for sexual development and meiosis are transcription factors (Chu *et al.* 1998; Kassir *et al.* 1988, 2003; Primig *et al.* 2000; Enyenihi and Saunders 2003). Our earlier study revealed 15 transcription factor genes that regulate aspects of sexual development in *N. crassa*, and mutation of 13 of these genes resulted in a complete block in ascospore production (Colot *et al.* 2006). Work from other laboratories has identified genes encoding the transcription factors *asm-1*/NCU01414 (Aramayo *et al.* 1996), *asd-4*/NCU15829 (Feng *et al.* 2000), *pp-1*/NCU00340 (Li *et al.* 2005), and *tan spore* [*ts*/NCU01459; (McCluskey *et al.* 2011)] all of which influence sexual development.

The collection of 242 viable transcription factor mutants was screened for the number, size, and other properties of protoperithecia (Figure 4A and Table S1). For perithecia, the mutants were assayed for abundance, relative size, and the presence of beaks. Ascospore ejection was scored by visual inspection of spores on the sides of the glass culture tube. We identified 47 mutants with at least one type of sexual cycle phenotype, corresponding to 19% of the viable mutants (Figure 1, Figure 4A, and Table 1). The C2H2 class had the largest number of genes with a sexual cycle phenotype (12 mutants; Figure S1 in File S1 and Table 1). Other transcription factor classes with a large proportion of mutants possessing a sexual development defect include WD40 (2/3 with phenotypes; 67%) and MYB (8/13 with phenotypes; 62%; Figure S1 in File S1 and Table 1).

Of the 47 mutants with a defect, 26 exhibited a complete block at a step in sexual development, with no development of ascospores (Figure 4A). A total of nine mutants were completely blocked in protoperithecial development (Figure 4A). Another 10 mutants produced protoperithecia, but no perithecia or ascospores, for a total of 19 mutants that did not form perithecia (Figure 4A). An example of a mutant that produces protoperithecia, but no perithecia, is *fmf-1*/NCU09387 (Figure 4B). Seven mutants elaborated protoperithecia and perithecia, but no ascospores (Figure 4A). In addition to those with a complete block at a step in sexual development, we identified numerous mutants with a reduction in the relative quantity of sexual structures (Figure 4A). There were 16 mutants affected in the number of protoperithecia, 12 in perithecial production, and 15 with fewer ascospores (Figure 4A). A majority of ARID (2/3; 67%), WD40 (2/3; 67%), and MYB (8/13; 62%) class mutants are affected in sexual development (Table 1).

Several mutants displayed more unique defects during the sexual cycle. Similar to previous results, the *ts*/NCU01459 mutant (McCluskey *et al.* 2011) produced tan ascospores (Table S1). The GATA mutant *sub-1*/NCU01154 produced protoperithecia that were submerged in the agar. Six different transcription factor families contribute to proper perithecial beak development; the homeodomain mutant *bek-1*/NCU00097 (Krystofova and Borkovich 2006), the C6 mutant *bek-2*/NCU07139 , the BHLH mutant *vsd-3*/NCU08999 and the BZIP mutant *ada-1*/NCU00499 completely lacked perithecial beaks, while another four mutants had a reduced number of perithecial beaks (C2H2 genes *cre-1*/NCU08807 and *vsd-9*/NCU07952, BHLH *dsd-3*/NCU05970, and APSES *vsd-5*/NCU07587; Table S1). The beak defect of *vsd-5* is presented in Figure 4B.

### Correlation between phenotypes and gene expression during sexual development

We took advantage of a publicly available dataset for a time course during perithecial and ascospore development (Wang *et al.* 2014) to investigate expression of transcription factor genes. This dataset includes eight time points (0, 2, 24, 48, 72, 96, 120, and 144 hr) after fertilization with opposite mating type wild type. Time = 0 corresponds to nitrogen-starved vegetative hyphae and unfertilized protoperithecia just before application of wild type conidia. Perithecia are obvious at the 24 hr time point, while croziers (evidence of meiosis) appear at 48–72 hr. Asci containing the meiotic progeny are formed after 96 hr, and perithecial beaks at 120–144 hr.

We first interrogated the data for transcription factor genes that yielded a sexual cycle phenotype (Figure 4C). There were several genes for which phenotypes and gene expression showed a correlation (Figure 4C). For example, Group 1 genes, with expression peaking early from 0 to 2 hr, included 6/9 mutants with defects in protoperithecial development, and 100% with perithecial phenotypes. The expression pattern and high proportion of perithecial defects suggests roles in fertilization for this group. Group 3 genes peak at 48 hr (time of meiosis), with 100% of the mutants possessing an ascospore defect. Group 4 genes are highly expressed from 72 to 144 hr and 4/5 of the corresponding mutants have a defect in perithecial formation and ascospore development/shooting. The large Group 6 is generally highly expressed late (120–144 hr), with 11/14 and 12/14 of the mutants exhibiting defects in perithecial and ascospore development, respectively. The more heterogeneously expressed Group 2 and 5 genes did not follow strict expression patterns, but all but one gene yielded a protoperithecial phenotype.

We next mined the expression data to identify cotranscribed genes in the same class of transcription factors (Figure S2 in File S1). This analysis revealed several instances of similarly expressed genes where one produced a moderate (nonblocking) sexual cycle phenotype, and the second a nonblocking defect or no phenotype, suggesting possible redundancy. The MYB genes *rca-1*/NCU01312 (no sexual phenotype) and *svd-4*/NCU09329 (severely reduced ascospores) are expressed at lower levels from 0 to 48 hr, with steady, elevated expression from 72 to 144 hr (Figure S2A in File S1), suggesting redundant roles late during sexual development. The C2H2 genes *svd-3*/NCU06487 (reduced number of ascospores) and *vad-12*/NCU05035 (no sexual phenotype) are highly expressed at 120–144 hr, the time of ascospore production (Figure S2B in File S1). The C2H2 genes NCU02487 (no sexual phenotype) and *svd-3*/NCU06487 (reduced number of ascospores) are highly expressed in unfertilized protoperithecia (*t* = 0), and from 120 to 144 hr, when ascospores are produced (Figure S2B in File S1). The C2H2 genes *vsd-9*/NCU07952 (reduced ascospores) and NCU03699 (no sexual phenotype) are coordinately expressed, with a dip at 48 hr (time of meiosis) and 144 hr (ascospore maturation) (Figure S2B in File S1). The C6 class genes *vsd-4*/NCU01243 (reduced number ascosopores) and *acu-15*/NCU06656 (normal sexual development) are both highly expressed in protoperithecia, and then peak again from 24 to 48 hr, at the time of meiosis (Figure S2C in File S1).

### Associations between phenotypes during different phases of the lifecycle

We identified a total of 154 mutants with at least one phenotype during growth or development (64% of viable mutants; Table 1). Inspection of mutants with two defects revealed 34 with growth and asexual development phenotypes, 12 with growth and sexual development defects, and six with phenotypes in asexual and sexual development, totaling 21% of the viable mutants (Figure 2 and Table 1). There were 19 strains with defects in all three stages assayed (growth, asexual development, and sexual development), comprising 4.1% of the mutants (Figure 2 and Table 1). Of interest, the majority of mutants with a conidiation defect also exhibited reduced hyphal growth rate (27/92 mutants; 82%). A lesser proportion also possessed sexual cycle defects (17/28 mutants; 61%). These observations suggest a possible linkage between the ability to produce conidia and growth rate and/or sexual development.

As our quantitative data for growth rate and aerial hyphae height were both obtained using vegetative (asexual) cultures, we investigated a possible relationship between these two phenotypes in our entire population of mutants (Figure 3C). Visual inspection of the plot did not reveal a clear correlation, and regression analysis of a line drawn through the global data had an *R*^2^ value of only 0.165 (Figure S3 in File S1), not supporting a linear relationship between growth rate and aerial hyphae height. When we restricted the data to mutants with a defect in at least one trait, the *R*^2^ value dropped to 0.144 (data not shown). Therefore, the growth rate of basal hyphae and aerial hyphae height appear to be independent traits in our group of transcription factor mutants.

We also investigated phenotypes for the group of 24 WCC-regulated transcription factor genes (Smith *et al.* 2010). We were most interested in whether two major phenotypes that are known to be light regulated—asexual and sexual development—were prevalent in this group of genes. Three of the genes did not pass our requirements to be classified as transcription factors (NCU00275, NCU06534, and NCU07846). Of the remaining 21 genes, we have complete phenotypic data for 18 mutants (Table S1), while phenotypes for another mutant (*ve-1*/NCU05964) have been published (Bayram *et al.* 2008). Nine of these 19 mutants have defects in asexual sporulation, two have both asexual and sexual sporulation phenotypes, and three mutants have only sexual cycle defects, for a total of 14 mutants (74%) with a sporulation phenotype (Figure S4A in File S1). Of interest, two of the mutants that lack phenotypes in asexual or sexual sporulation (NCU07705 /*clr-1* and NCU08000 ) are similarly expressed during sexual development (Figure S4B in File S1; neighbors on the heat map), suggesting possible redundancy during this process. Taken together, our results mesh well with the findings of the earlier study (Smith *et al.* 2010), and support these transcription factors as potential second-level regulators in the blue light gene expression hierarchy that regulates asexual and sexual development in *N. crassa*.

## Discussion

We have analyzed phenotypes in available mutants for annotated transcription factor genes in *N. crassa*. Overall, 64% of the mutants analyzed possessed at least one growth or developmental phenotype. The largest proportion of mutants exhibited defects in hyphal growth rate, either alone or in combination with another defect. This result underscores the importance of transcriptional regulation to the various steps of hyphal growth, including spore germination, hyphal growth and polarity, branching, and anastomosis. The latter two phenomena are often accentuated in slow-growing mutants (so-called colonials; Brody and Tatum 1966; Kolmark 1969) and this may contribute to the slow growth rate of some of the mutants in our study.

Inspection of data for individual transcription factor classes revealed several with a large number of genes with defects and/or skewing toward specific defects. For transcription factor classes represented by more than two mutants, 100% of the ARID/BRIGHT, NDT80 and WD40 mutants possessed phenotypes. The majority (83%) of GATA factor mutants possessed defects in asexual development, while 75% of the BHLH and 62% of the MYB domain mutants had a growth rate phenotype. There was less concentration of sexual development defects in certain classes, with WD40 (67%) and MYB (58%) having the largest proportion of mutants with sexual cycle phenotypes.

Comparison of our current data with our original analysis of 99 viable transcription factor mutants revealed several phenotypes that were greatly under-represented in the earlier study. For example, there has been a fivefold increase in mutants with only a basal hyphae growth rate phenotype, and a fourfold increase in mutants with defects in both growth rate and asexual development. There were zero and one mutants with hyphal growth/sexual development and asexual development/sexual development phenotypes, respectively (Colot *et al.* 2006); there are now 12 mutants in the first group and six mutants in the second (Figure 2). In general, the more than doubling of the number of mutants analyzed has resulted in discovery of more genes that affect basal hyphae growth and asexual development. This difference also contributes to the increase in the relative number of genes that yield phenotypes, from 40% (Colot *et al.* 2006) to 64% (this study).

We have previously applied our phenotypic screening platform to other large gene families in *N. crassa*, including serine-threonine protein kinases [77 mutants; (Park *et al.* 2011b)], serine-threonine protein phosphatases [24 mutants; (Ghosh *et al.* 2014)] and G protein coupled receptors [GPCRs; 36 mutants; (Cabrera *et al.* 2015)]. The percentage of transcription factor mutants with at least one growth/developmental phenotype (64%) is less than that for the protein phosphatases (91%), but greater than that observed for GPCRs (47%) and protein kinases (57%). Comparison between these groups reveals that the proportion of mutants with hyphal growth rate defects is relatively low for GPCRs (14%), but similar for the protein kinase (42%), transcription factor (43%), and protein phosphatase (50%) genes. In the case of asexual sporulation, the fraction of affected mutants was identical/near identical for transcription factors (38%), GPCRs (39%) and protein kinases (40%), but significantly higher for protein phosphatases (58%). For sexual development, GPCRs and transcription factors had a much lower proportion of genes with phenotypes (17 and 19%, respectively) than the protein kinases (42%) and protein phosphatases (63%). There were also striking differences in the percentage of mutants with defects in all three categories among the four groups, from GPCRs with no such mutants, to 8% of transcription factor, 26% of protein kinase, and 29% of protein phosphatase mutants. The lower number of transcription factor and GPCR mutants with sexual development defects or phenotypes in all three categories analyzed may reflect greater gene redundancy in transcription factors and GPCRs. We hypothesize that there may be greater functional redundancy in genes at the opposite ends of the environmental sensing spectrum (receptors and transcription factors), than with those more involved in signal transduction and integration (kinases and phosphatases). This may be more obvious during sexual development due to the large number of cell types involved (Bistis *et al.* 2003), and the need to coordinate cell morphogenesis with meiosis. Alternatively, the greater apparent redundancy may reflect functions for receptors and transcription factors that are not currently being analyzed in our phenotypic assays. Testing of these and other alternative hypotheses will require further investigation.

Transcription factor genes have been annotated in several other filamentous fungal species. In the genus Trichoderma, the biological control agents and mycoparasites *Trichoderma virens* and *Trichoderma atroviride*, and the efficient cellulose degrader and industrial species *T. reesei* have 641, 592, and 448 transcription factor genes, respectively (Schmoll *et al.* 2016) (5.1, 5.0, and 4.9% of the genes in each genome). The two mycoparasitic species exhibited a large expansion in the C6 zinc cluster transcription factor family, with 382 genes for *T. atroviride* and 422 for *T. virens*, compared to 258 in *T. reesei*. In *Aspergillus nidulans*, there are 490 annotated transcription factors, corresponding to 4.6% of the predicted genes (Wortman *et al.* 2009). Similar to Trichoderma species, *A. nidulans* has significantly more C6 genes than *N. crassa* [330 genes; (Wortman *et al.* 2009)]. A more recent study reported the number of C6 zinc cluster transcription factor genes in three Aspergilli species, with 180 in *A. clavatus*, 276 in *A. nidulans*, and 306 in *A. flavus* (Chang and Ehrlich 2013). In contrast to C6 proteins, the number of C2H2 proteins reported for other species is similar to, or even less than in, *N. crassa*, with 60 in *A. nidulans* (Wortman *et al.* 2009), 61 in *T. virens*, 53 in *T. atroviride*, and 49 in *T. reesei* (Schmoll *et al.* 2016). Thus, the larger number of C6 proteins in these other fungi is not due to a proportional increase in all transcription factor families. Rather, it appears that the expansion of the fungal-specific C6 class is a major contributor to the greater number of transcription factors overall in Trichoderma species and *A. nidulans* relative to *N. crassa*. The reduced size of the C6 class in *N. crassa* may result, at least in part, from Repeat-Induced Point Mutation (RIP), a mechanism that mutates duplicated DNA sequences during the sexual cycle and that has been hypothesized to limit the size of gene families in the *N. crassa* genome (Galagan *et al.* 2003; Selker 1990).

In this study, we investigated functions for 242 predicted transcription factor genes during growth and asexual and sexual development in *N. crassa*. Considering the significant sample size and the accompanying penchant for gene redundancy, our results revealed a surprisingly large proportion of mutants with at least one phenotype. We identified numerous genes that lack sexual cycle phenotypes that are coordinately expressed with other genes with nonblocking phenotypes during sexual development. This suggests that these genes possess overlapping functions during sexual differentiation, a hypothesis that can be addressed in future experiments through construction of mutants lacking multiple transcription factor genes. This work has augmented our knowledge of the functions of transcription factors during growth and development in filamentous fungi.

## Supplementary Material

Supplemental material is available online at www.g3journal.org/lookup/suppl/doi:10.1534/g3.117.043331/-/DC1.
