# Supplementary material for: Functional Profiling of Transcription Factor Genes in Neurospora crassa
Source: G3 (Bethesda). 2017 Jul 7;7(9):2945–56. doi: 10.1534/g3.117.043331 (PMC5592922; doi:10.1534/g3.117.043331)
Supplement: Supplementary file 1 [file 2945FileS1.pdf]

SUPPLEMENTARY FIGURES

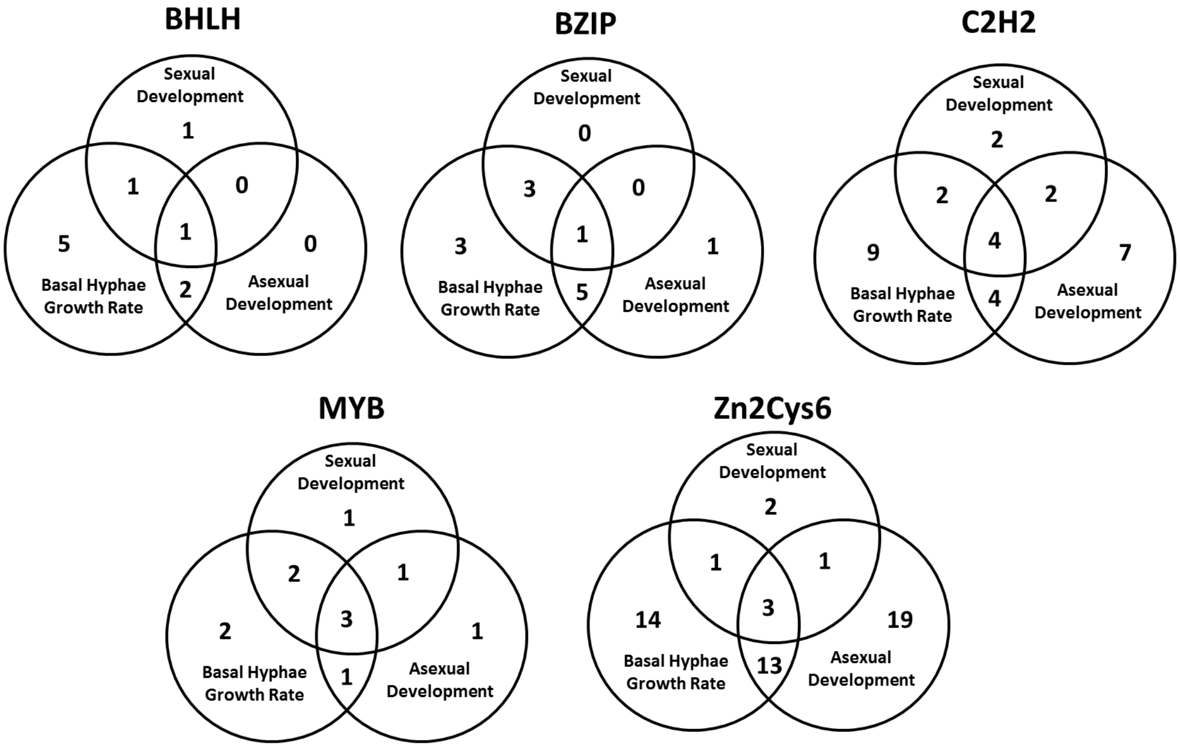

Figure S1. Phenotype summary for major transcription factor classes. The total number of mutants with the indicated defect or combination of defects is shown in each lobe of the Venn diagram for that transcription factor class.

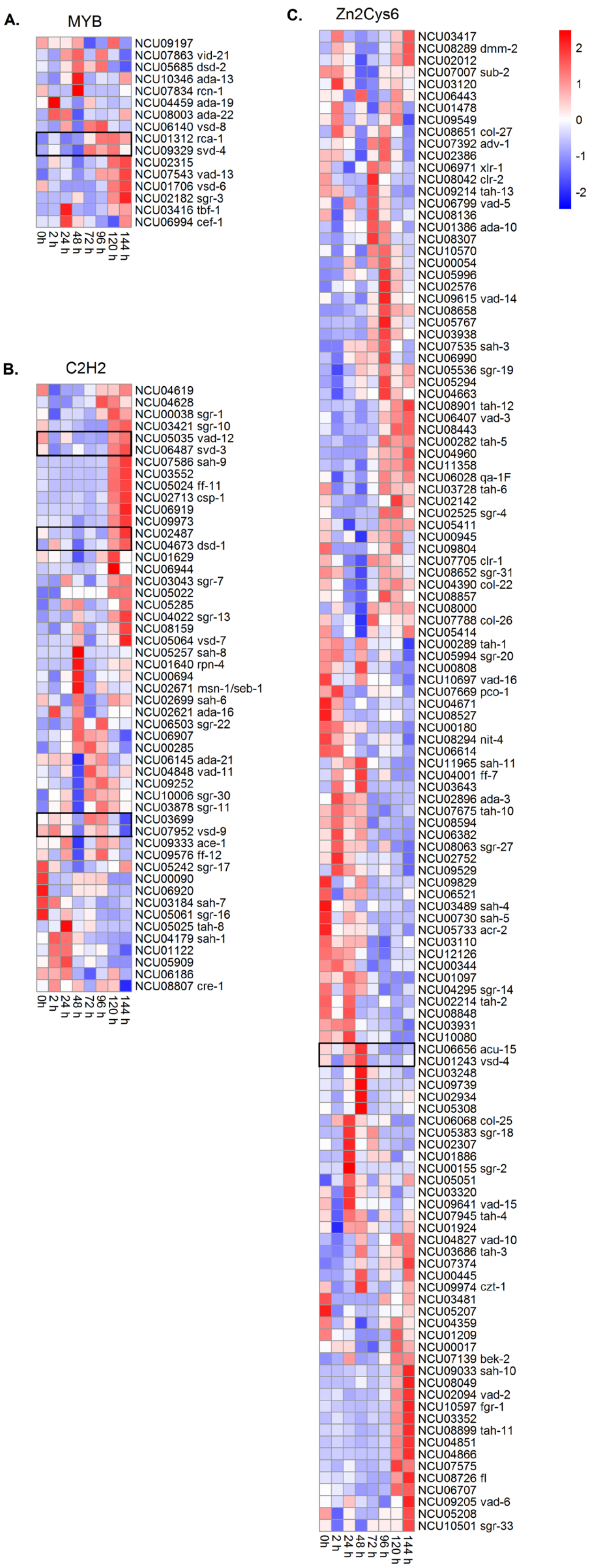

Figure S2. Sexual development time course heat maps for MYB, C2H2 and Zn_2Cys_6 (C6) transcription factor classes. The clustering and heatmap analysis presented in Figure 4C was performed on all mutants in each of the displayed transcription factor classes, whether or not they had a sexual development phenotype. Boxed groups of genes include one gene with a non-blocking sexual development phenotype and another gene with no phenotype or a non-blocking defect that is similarly expressed during sexual development. The values for each gene are normalized to the RPKM at the time point with lowest expression for that gene.

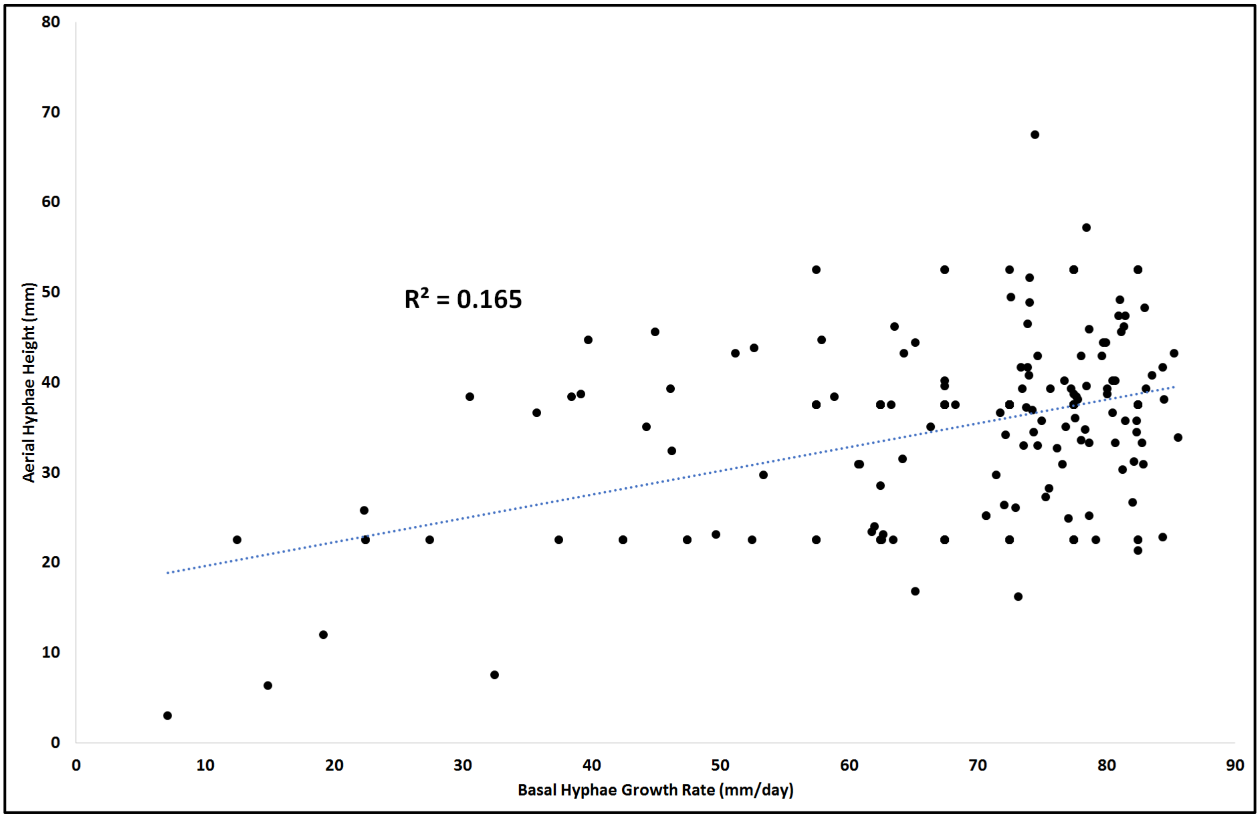

Figure S3. Linear regression analysis of aerial hyphae height and hyphal growth rate data. Data are taken from Figure 3C. The R^2 value for the line drawn through the data has a value of 0.165.

A.

Gene Number	Gene Name	Class	Asexual Development	Sexual Development
NCU01871		BHLH	***N/A***	***N/A***
NCU04731	*sah-2*	BHLH	AH	
NCU02713	*csp-1*	C2H2	AH	
NCU03184	*sah-7*	C2H2	AH	
NCU08159		C2H2	***N/A***	***N/A***
NCU08807	*cre-1*	C2H2	C	P,A
NCU06095	*csp-2*	CP2	AH	
NCU01154	*sub-1*	GATA		PP,P,A
NCU07728	*sre*	GATA	AH	
NCU09068	*nit-2*	GATA	AH	
NCU00097	*bek-1*	Homeodomain		P,A
NCU05964	*ve-1*	Velvet	*	
NCU01243	*vsd-4*	Zn2Cys6		A
NCU02094	*vad-2*	Zn2Cys6	AH	
NCU04295	*sgr-14*	Zn2Cys6		
NCU05994	*sgr-20*	Zn2Cys6		
NCU07392	*adv-1*	Zn2Cys6	AH,C	PP,P,A
NCU07705	*clr-1*	Zn2Cys6		
NCU08000		Zn2Cys6		
NCU09615	*vad-14*	Zn2Cys6	AH	
NCU09829		Zn2Cys6		

B.

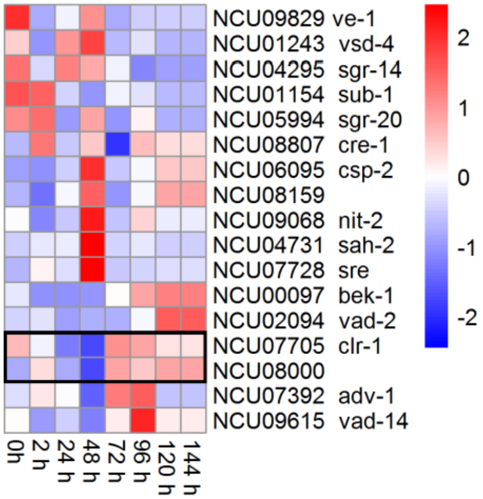

Figure S4. Asexual/sexual sporulation phenotypes and sexual development expression data analysis for transcription factors regulated by the White Collar Complex (WCC).
A. Asexual and sexual development phenotype summary. Asexual sporulation (aerial hyphae height and/or conidia production) and sexual development (protoperithecia, perithecia and ascospore production) defects for available mutants lacking 18 transcription factors that are regulated by the WCC (see (Smith et al. 2010)). *Data supporting an asexual sporulation defect for *ve-1* mutant taken from (Bayram et al. 2008).
B. Sexual development time course heat map. Available RNAseq data for 17/21 transcription factor genes regulated by the WCC were subjected to clustering and heatmap analysis as described in Figure 4C. NCU0775/*clr-1* and NCU08000 (boxed) lack sexual cycle phenotypes, but are coordinately expressed during sexual development.

References for Supplementary Figures

Bayram, O., S. Krappmann, S. Seiler, N. Vogt, and G.H. Braus, 2008 *Neurospora crassa ve-1* affects asexual conidiation. *Fungal Genet Biol* 45 (2):127-138.

Smith, K.M., G. Sancar, R. Dekhang, C.M. Sullivan, S. Li *et al.*, 2010 Transcription factors in light and circadian clock signaling networks revealed by genomewide mapping of direct targets for Neurospora White Collar Complex. *Eukaryot Cell* 9 (10):1549-1556.
